# Four night eating behavior subtypes in young and middle-aged adults with type 2 diabetes: insights for targeted public health interventions

**DOI:** 10.3389/fpubh.2026.1718149

**Published:** 2026-02-11

**Authors:** Lanlan Yu, Hantian Cheng, Yuehua Qin, Lanlan Zhou, Yuan Zhou

**Affiliations:** 1 Department of Endocrinology, The First Affiliated Hospital with Nanjing Medical University, Nanjing, Jiangsu, China; 2 Department of Nursing, The Fourth Affiliated Hospital of Soochow University, Suzhou, Jiangsu, China; 3 Department of Neurosurgery, The First Affiliated Hospital with Nanjing Medical University, Nanjing, Jiangsu, China

**Keywords:** behavior subtypes, night eating behavior, qualitative study, targeted public health interventions, type 2 diabetes mellitus, young and middle-aged adults

## Abstract

To address the limited evidence for public health interventions targeting night eating behavior (defined as eating between dinner and bedtime or waking to eat during sleep) in young and middle-aged T2DM patients in China, this qualitative interview study explored their real-life experiences, motivations, and influencing factors. Using the FBM, we identified night eating characteristics and developed corresponding behavioral subtypes to inform targeted public health strategies. From October to December 2024, 15 eligible patients were recruited from a tertiary grade A general hospital in China. Semi-structured interviews were conducted, and data analyzed via Colaizzi’s 7-step method. This study elucidated the dynamic night eating process, encompassing pre-eating triggers, psychological conflicts, emotional changes during eating, post-eating experiences, and subsequent coping behaviors. Six core influencing factors were identified (motivation, ability, triggers, dietary habits, psychological traits, sociocultural influences), and based on these, four subtypes were classified: “physiological stress-driven,” “emotional and psychological-driven,” “social environment-induced,” and “health cognitive bias-driven.” The findings provide a localized, targeted basis for community diabetes management (e.g., subtype-specific screening tools, family-collaborative interventions). Furthermore, this study broadens the FBM’s application in chronic disease behavior research and offers refined directions for personalized public health education and comprehensive disease management. It adhered strictly to the EQUATOR Network guidelines, an internationally recognized framework for reporting health research.

## 1 Introduction

This study focuses on young and middle-aged patients with type 2 diabetes mellitus (T2DM), a group accounting for 59% of China’s T2DM population (18–59 years old) and the fastest-growing subgroup in terms of prevalence (1). According to the International Diabetes Federation (IDF), China had the world’s largest diabetic population by 2021, with T2DM representing over 90% of cases (2). As primary bearers of family and social responsibilities, these individuals face high work pressure and frequent social engagements—factors contributing to a high prevalence of night eating behavior (defined as supplementary eating from dinner to bedtime or waking up to eat during sleep, excluding medically necessary midnight snacks like those for hypoglycemia) (3). Night eating not only leads to weight gain and sleep disorders but also increases blood glucose control difficulty, elevating the risk of diabetes-related complications (4, 5)-a critical issue given this group’s irregular lifestyles and heightened psychological stress (6).

However, current research on T2DM dietary management has three critical limitations: (1) it predominantly focuses on daytime dietary optimization, neglecting night eating (7); (2) most adopt a “homogeneous” analytical framework, lacking subdivision of specific behaviors like night eating; (3) quantitative studies (focused on statistical correlations) fail to capture subjective experiences, psychological motivations, and multi-dimensional triggers of night eating, hindering a comprehensive understanding of its mechanisms (i.e., intrinsic pathways of night eating) (e.g., insufficient exploration of causative factors for this subgroup). These gaps highlight the need for further targeted research.

To address this, we incorporate the Fogg Behavior Model (FBM), proposed by B. J. Fogg (8), which dissects behavior into three core components—motivation (willingness to perform the behavior), ability (ease of performing it), and trigger (external/internal cues initiating it)-and is widely used in health-related behavior research (9). For clarity, we define “comprehensive behavioral subtypes” as a classified description of behavioral characteristics integrating lived experiences, influencing factors, and theoretical components (e.g., FBM) to distinguish behavior subtypes (10).

The core logic of this study adheres to the sequence of “first exploring lived experiences, then constructing behavioral subtypes.” Its primary objective is to investigate the intrinsic motivations, in-depth lived experiences, and multi-dimensional influencing factors (psychological, physiological, social) underlying night eating behavior among young and middle-aged patients with T2DM, laying a phenomenological foundation for subtype construction. The secondary objective is to develop comprehensive night eating behavioral subtypes by integrating the FBM with empirical findings from experience exploration, thereby providing evidence-based support for personalized interventions. This study seeks to establish experience-grounded behavioral subtypes. To achieve this, we address four research questions: (1) What are the intrinsic motivations for night eating behavior? (2) What constitutes the in-depth lived experiences of night eating? (3) What multi-dimensional factors influence night eating behavior? (4) What behavioral subtypes can be constructed based on lived experiences and the FBM?

## 2 Materials and methods

### 2.1 Design

This study utilized phenomenological qualitative research methods (11) to investigate the characteristics and influencing factors of night eating patterns in young and middle-aged individuals with type 2 diabetes mellitus. Semi-structured interviews were conducted to gather data. The interview protocol was developed based on the FBM, focusing on key elements of the model such as motivation, ability, and triggers. This approach aimed to systematically capture information on patients’ intrinsic motivation, ability to implement behaviors, and trigger cues related to night eating, facilitating a comprehensive exploration of behavioral mechanisms within the model framework. Adherence to the reporting guidelines outlined in the Consolidated Criteria for Reporting Qualitative Research (COREQ) (12) ensured the standardized presentation of qualitative study findings.

### 2.2 Study setting and recruitment

This study adopted a purposive sampling method to enroll individuals diagnosed with type 2 diabetes mellitus (T2DM) who were admitted to the Department of Endocrinology of a Grade A tertiary hospital in Nanjing, Jiangsu Province. The inclusion criteria were as follows: (1) meeting the diagnostic criteria for T2DM specified in the *China Guidelines for the Prevention and Treatment of Type 2 Diabetes* (latest officially recommended version) (13); (2) being young and middle-aged adults; (3) having the habit of night eating, specifically eating between dinner and bedtime or waking up to eat during sleep; (4) being conscious, capable of effective communication, and providing written informed consent. The exclusion criteria included: (1) having type 1 diabetes mellitus, gestational diabetes mellitus, severe liver or kidney dysfunction, cancer, other life-threatening illnesses, mental disorders, hearing impairments, or intellectual disabilities; (2) being unable to cooperate with interviews or having communication disorders. (3) with confirmed eating disorders, such as anorexia nervosa, bulimia nervosa, and binge-eating disorder, or related abnormal behaviors, including binge eating and excessive dieting, as determined by standard admission assessments and medical records. To enhance the rigor of participant selection, the inclusion/exclusion criteria were reviewed by two nursing experts specializing in chronic disease management to ensure they were operational and aligned with the research objectives. This helped avoid ambiguous eligibility judgments during recruitment.

Initially, 40 patients with night eating behavior were screened, of whom 10 were excluded (4 with type 1 diabetes, 3 declining interviews, 3 with age mismatch), leaving 30 eligible patients. We adopted consecutive recruitment of these 30 eligible patients to minimize selection bias, and 15 participants with heterogeneous night eating behaviors were finally selected from them in line with qualitative research requirements for the study.

Of the 15 participants, 73.3% (n = 11) were hospitalized for intensive glycemic control due to poor HbA1c (≥8.0%), a common clinical presentation in this population.

The sample size was determined based on the principle of data saturation, as defined by Saunders et al. (14)—a point at which no new themes emerge from the data. After reaching the initial saturation (i.e., no new themes were identified from the 15th participant), three additional participants were interviewed consecutively to verify saturation, and no new themes emerged in these interviews. Thus, the final sample size was determined to be 15 participants.

### 2.3 Data collection

Data were collected through two complementary methods: blood glucose data collection and semi-structured interviews, to ensure the comprehensiveness and reliability of research data.

#### 2.3.1 Blood glucose data collection

For glucose variability (GV) assessment, all participants uniformly underwent continuous glucose monitoring (CGM, Yuwell CT2) during hospitalization for no less than 72 h (maximum 7 days, per the device’s limits), with glucose levels recorded every 3 min. For consistent and standardized GV calculation, the first 72 h of valid CGM data were uniformly extracted. GV was computed as the coefficient of variation (CV), using the formula [(standard deviation/mean glucose) × 100%], and all results were uniformly reported in this study.

#### 2.3.2 Semi-structured interview data collection

Based on the Fogg Behavior Model (B = MAP), an interview framework was constructed around three core elements: motivation, ability, and cue. An initial interview outline was developed based on the research objectives and literature review; it was then revised and finalized by the research team through group discussions after consulting two qualitative research experts and conducting pre-interviews with three participants (see Table 1). Each interview lasted approximately 10–20 min.

**Table 1:** Semi-structured interview guide.

Question no.	Interview questions	Relevant dimension (Fogg behavior model)
1	Did your nighttime eating behavior occur before or after being diagnosed with diabetes?	Motivation
2	Under what circumstances do you engage in nighttime eating? Please discuss the following aspects (dimensions: emotions, physiological factors, food-related factors, sleep quality, preferences, environmental factors, social factors, dietary habits, health beliefs, cultural customs).	Motivation, ability, trigger
3	After nighttime eating, what specific physical sensations do you experience? How does your emotional state change?	Motivation
4	Do you think nighttime eating affects your blood sugar control? Why do you continue this behavior despite its potential impact?	Motivation
5	What types of food do you typically consume during nighttime eating? Approximately how much do you eat?	Ability
6	Do you believe you can control your nighttime eating behavior? If not, what are the main challenges? Additionally, if you want to adjust your nighttime eating behavior, what kind of support or help do you think you need the most?	Ability
7	At what time during the night do you usually eat? How frequently does this occur? Additionally, what activities or behaviors are you typically engaged in within 1 h before you have a nighttime meal?	Trigger
8	What are the attitudes of your family and friends toward your nighttime eating behavior? How does this influence you?	Trigger
9	What thoughts or circumstances might discourage you from nighttime eating? Have you read any health education articles about the impact of nighttime eating on type 2 diabetes? If so, how have these articles influenced you?	Trigger

To ensure data quality, the initial interview outline was co-reviewed by two nursing experts (with qualitative research expertise) and a behavioral science psychologist, confirming its validity and alignment with the research focus. All interviews were professionally recorded, underwent standardized transcription immediately, and verified via a double cross-check mechanism (one researcher checked transcript-recording consistency; another cross-referenced with participants’ self-reports) to minimize transcription errors. Additionally, subjective data (e.g., self-reported feelings) were cross-referenced with objective clinical data (e.g., blood glucose records, medication adherence) to enhance reliability.

To protect privacy, participants were assigned pseudonyms (N1 to N15) in all interview records and data analyses. All participants voluntarily signed written informed consent prior to interviews (consistent with the inclusion criterion in Section 2.3).

### 2.4 Ethical considerations

This study strictly adhered to the *Declaration of Helsinki* (15)and was endorsed by the affiliated hospital of a medical university which strictly adheres to ethical principles to protect the privacy of the respondents (approved by the Ethics Committee of The First Affiliated Hospital with Nanjing Medical University).

Prior to study enrollment, all participants were fully informed of the research objectives, procedures, voluntary participation rights (including the right to withdraw at any time without reason), and confidentiality measures. Only those who provided explicit consent signed written informed consent forms.

To ensure confidentiality: (1) Participants were identified by pseudonyms instead of real names in all research materials; (2) All electronic data (including transcripts and audio recordings) were encrypted and stored on a password-protected server, with access restricted exclusively to the core research team; (3) Personal identifiers that might enable identification (e.g., age, occupation) were excluded from the final analysis and manuscript. To facilitate research reproducibility, all original data (including recordings, transcripts, and clinical records) were meticulously organized, numbered, and encrypted for secure archiving. This allowed for methodological verification and potential re-analysis if needed.

### 2.5 Rigor

This study improved research rigor and reduced bias through a triangulation strategy.

For data triangulation, the subjective data of participants (e.g., self-reported feelings) were cross-referenced with objective clinical data (e.g., blood glucose records), which reduced information bias caused by reliance on a single data source and improved the reliability of results.

For method triangulation, the interview outline was reviewed by two nursing experts specializing in chronic diseases (to ensure the validity of the research instrument); combined with professional recording, standardized transcription, and a double cross-check mechanism (to ensure data accuracy), the entire study was reviewed in accordance with the Consolidated Criteria for Reporting Qualitative Research (COREQ) 32-item Checklist (to ensure methodological consistency), which reduced bias in research design and implementation.

Original data were systematically organized, numbered, encrypted, and archived to ensure reproducibility. Contextual information including participants’ age and disease duration was provided in Table 2 to improve the transferability of the study, helping readers evaluate the generalizability of the results.

**Table 2:** Characteristics of patient participants—demographics and disease duration (*N* = 15).

Variables	Frequency	Percent
Gender		
Female	8	53.3
Male	7	46.7
Age		
18–45 years	10	66.7
46–59 years	5	33.3
Marital status		
Single	3	20.0
Married	12	80.0
Occupation		
Office worker	4	26.7
Freelancer	3	20.0
Self-employed	2	13.3
Student	1	6.7
Worker	1	6.7
Retired	1	6.7
Enterprise manager	1	6.7
Homemaker	2	13.3
Educational background		
Undergraduate	5	33.3
College Diploma	4	26.7
Primary School	3	20.0
Junior High School	1	6.7
High School	1	6.7
Graduate Student	1	6.7
Disease duration		
Newly diagnosed(≤30 days)	7	46.7
Chronic phase(>30 days)	8	53.3

BMI: Body Mass Index, weight (kg)/height^2^(m). HbA1c: Glycated hemoglobin, reflects 2–3 – month average blood glucose. CV of blood glucose: Coefficient of Variation, used as an indicator of blood glucose variability (GV) to reflect the degree of blood glucose fluctuation. HOMA-IR: Homeostatic Model Assessment – Insulin Resistance, based on fasting glucose and insulin, unitless.

### 2.6 Data analysis

This study adopted Colaizzi’s seven-step analysis method (16) for qualitative data interpretation and theme extraction. Meanwhile, the FBM was employed as the theoretical framework to guide the subtype classification of night eating behavior. On this basis, the research team systematically categorized the coded content according to the “Motivation-Ability-Trigger (MAT)” framework during the theme induction process.

First, based on the phenomenological principle of “extracting core experiences,” criteria for determining dominant motivation were established: ① All expressions related to night eating motivation were extracted from the verbatim interview transcripts, with a focus on three types of statements: those mentioned repeatedly (frequency ≥ 3 times), those with strong emotional overtones, and those explicitly described by the interviewees as the “determinant cause” of their behavior; ② A motivation was identified as the dominant one only if it met the three conditions simultaneously: high-frequency mention, high emotional intensity, and explicit behavioral driving effect.

Second, two nursing researchers with more than 5 years of qualitative research experience and systematic training in phenomenological analysis independently conducted open coding. In case of coding discrepancies between the two researchers, they first elaborated their respective coding rationales, then cross-verified the original interview data (including audio recordings and verbatim transcripts). If the discrepancies remained unresolved, a third-party researcher (a nursing expert with 10 years of experience in qualitative research on chronic diseases) was invited to participate in the review until a consensus was reached. After resolving all discrepancies, Cohen’s kappa coefficient was recalculated for all coding results, with a final kappa value of 0.85, indicating good consistency of the coding outcomes.

Finally, the research team adopted thematic clustering and case comparison for subtype classification: participants with similar behavioral patterns were initially grouped into candidate categories; subsequently, representative interview segments were selected for each candidate category, and the descriptions of motivation intensity, triggering situations, etc., were thoroughly compared to verify the consistency of characteristics within each category and the discriminability between different categories.

For boundary cases with overlapping characteristics, the research team referred back to the original interview records and made a comprehensive judgment by combining the frequency of motivation expression, emotional intensity, and the significance of behavioral impact. Ultimately, after multiple rounds of group discussions and reverse validation against the FBM theoretical framework, the night eating behaviors of middle-aged and young research subjects with type 2 diabetes were classified into four subtypes (17): Physiologically Driven Type, Emotional-Psychologically Driven Type, Environmentally Induced Type, and Health Cognition Bias Type. Each subtype exhibited relatively stable FBM three-dimensional structural characteristics and showed differences in clinical manifestations and intervention needs.

To intuitively present the differentiated characteristics of night eating behaviors in the study cohort, this study adopted the attribute characteristic word extraction method for research subjects, using the extracted characteristic words as behavioral description indicators. Weights were assigned to these characteristic words based on their occurrence frequency and importance; a word cloud generation tool was then used to create visualized word clouds, supplemented by statistical tables to elaborate on the specific characteristics of night eating behaviors in this population.

### 2.7 Researcher reflexivity

This study was conducted by five female professionals with nursing and endocrinology backgrounds, with clear roles. Their core qualifications are as follows:

Lanlan Yu (First Author and Interviewer): Part-time nursing graduate student, Registered Nurse (RN), 8 years of endocrine diabetes care experience, 30-h qualitative interview training (2024), led interviews and data analysis.

Hantian Cheng (Co-First Author): Master of Nursing candidate, RN, diabetes nursing management training (2023), responsible for data collation and coding verification.

Yuehua Qin (Researcher): Part-time nursing graduate student, over 15 years of comprehensive endocrine nursing experience, proficient in patient needs assessment, participated in data validation and result interpretation.

Lanlan Zhou (Co-Corresponding Author): Part-time professional (equivalent nursing qualification), Associate Chief Nurse, more than 10 years of chronic disease nursing research experience, qualitative research training (2022), oversaw study design, methodological rigor, and manuscript final review.

Yuan Zhou (Co-Corresponding Author): Master of Nursing candidate, Nurse-in-Charge, 8 years of interdisciplinary research coordination experience, clinical research ethics training (2023), assisted in ethical approval and logistics.

Prior to the study, there were no personal or professional relationships between the researchers and the 15 participants. All participants were recruited from inpatients in the Department of Endocrinology, consistent with Section 2.2’s inclusion criteria, to mitigate potential relationship bias. During the informed consent process, participants were explicitly made aware of the researchers’ dual roles as clinical nurses in Endocrinology and independent research investigators, as well as the study’s purpose: exploring the night eating experiences of hospitalized young and middle-aged patients with type 2 diabetes. They were also informed that the interview content would be utilized exclusively for academic research, unrelated to clinical treatment or nursing evaluation, and that no personal information unrelated to the research would be disclosed, thereby ensuring the voluntary participation of all participants.

To mitigate response bias potentially arising from the clinician-patient role disparity in the inpatient setting, three quality control measures were implemented during the interviews. First, privacy control was established by conducting interviews in private consulting rooms or unoccupied wards, ensuring that no irrelevant personnel were present. This arrangement allowed participants to express their true feelings. Second, informed consent was confirmed by re-explaining the study’s purpose and the principle of voluntary participation in plain language. Participants were assured that pausing or terminating the interview would not impact their clinical treatment. Lastly, data validity was verified by inviting family members to join the interviews for cross-validation, with the participants’ consent. For participants with low cooperation, the interview was terminated, and relevant circumstances were documented to mitigate data distortion.

The interviewer (Lanlan Yu) developed research interest from clinical observations of patients’ night eating distress without preset assumptions. To control biases, her training emphasized neutral questioning; she maintained post-interview reflective journals, collectively reviewed by the team to avoid subjective guidance biases. All conclusions are derived from data of 15 participants, reflecting only this sample’s night eating experiences and potential associations. No generalization to all young and middle-aged T2DM patients is intended, with relevant limitations detailed in the Discussion section.

## 3 Results

### 3.1 General demographic and clinical characteristics of participants

Relevant details of the participants are presented in the following tables: Table 2 outlines their demographic characteristics and basic disease-related information, and Table 3 summarizes their clinical metabolic indicators (collected via standard clinical procedures during their current hospitalization), complications, and comorbidities.

**Table 3:** Characteristics of patient participants— clinical and metabolic parameters (*N* = 15).

*N*	BMI	Latest HbA1c	CV of Blood Glucose	HOMA-IR	Complication	Treatment Modality (Hospitalization)	Comorbidity
	(kg/m^2^)	(%)	(%)				
1	29.7	8.0	20.3	2.3	Type 2 diabetic nephropathy stage i (Mild), Type 2 diabetic peripheral neuropathy (Mild)	CSII: Continuous subcutaneous insulin infusion	Hypertension
2	25.6	10.2	22.6	1.4	Type 2 diabetic peripheral neuropathy (Mild), Type 2 diabetic peripheral vascular disease (Mild)	CSII: Continuous subcutaneous insulin infusion	Fatty liver
3	20.8	12.2	25.3	5.5	NO	CSII: Continuous subcutaneous insulin infusion	No comorbidities
4	26.3	10.6	25.0	4.0	NO	CSII: Continuous subcutaneous insulin infusion	Dyslipidemia
5	25.2	7.2	32.7	3.2	NO	CSII: Continuous subcutaneous insulin infusion	No comorbidities
6	24.2	9.1	27.3	7.7	Type 2 diabetic retinopathy (Mild), Type 2 diabetic nephropathy stage III (Moderate)	CSII: Continuous subcutaneous insulin infusion	Dyslipidemia, hypertension
7	20.3	10.6	28.6	2.5	NO	CSII: Continuous subcutaneous insulin infusion	Dyslipidemia, hypertension
8	25.3	6.3	28.4	2.6	Type 2 diabetic nephropathy stage I (Mild)	CSII: Continuous Subcutaneous Insulin Infusion	Dyslipidemia
9	28.4	9.7	15.8	2.6	NO	CSII: Continuous subcutaneous insulin infusion	Hypertension
10	27.3	10.7	32.7	4.9	NO	CSII: continuous subcutaneous insulin infusion	Fatty liver
11	26.9	11.1	35.3	1.3	NO	CSII: continuous subcutaneous insulin infusion	Hypertension
12	25.7	11.5	34.8	10.3	Type 2 diabetic autonomic neuropathy (Mild), Type 2 diabetic peripheral vascular disease (Mild)	CSII: continuous subcutaneous insulin infusion	No comorbidities
13	34.3	4.7	28.8	3.9	NO	CSII: Continuous subcutaneous insulin infusion	Obesity, dyslipidemia, fatty liver
14	28.7	6.1	28.4	6.7	NO	CSII: Continuous subcutaneous insulin infusion	Hypertension, liver dysfunction
15	23.9	9.7	25.4	13.5	Type 2 diabetic peripheral vascular disease (Mild), Type 2 diabetic retinopathy (Mild)	CSII: Continuous subcutaneous insulin infusion	No comorbidities

As presented in Table 2, all 15 participants were aged between 18 and 59 years, defined based on China’s legal retirement age of 60 years. Among these individuals, 11 were formally employed, while 2 were homemakers, representing 86.7% of the total. When stratified by disease duration, 7 participants (46.7%) were newly diagnosed (disease duration ≤ 30 days), and 8 participants (53.3%) were in the chronic phase (disease duration > 30 days).

As shown in Table 3, the glycated hemoglobin (HbA1c) levels among the participants varied from 4.7 to 12.2%, with 11 participants (73.3%) exhibiting HbA1c levels of 8.0% or higher. The glucose variability (GV) ranged from 15.8 to 35.3%, with 11 participants (73.3%) demonstrating GV values exceeding the normal range (< 25%). The homeostasis model assessment of insulin resistance (HOMA-IR) values ranged from 1.3 to 13.5, with 11 participants (73.3%) presenting HOMA-IR levels above the normal threshold (>2.5).

Six participants (40.0%) experienced mild to moderate diabetes-related complications, with some individuals having multiple complications. Specifically, there were 3 cases of diabetic peripheral vascular disease, 3 cases of diabetic nephropathy (including 2 cases of Stage I and 1 case of Stage III), 2 cases of diabetic retinopathy, 2 cases of diabetic peripheral neuropathy, and 1 case of diabetic autonomic neuropathy. Additionally, 6 participants (40.0%) had hypertension as a comorbidity, while 7 participants (46.7%) exhibited obesity-related metabolic abnormalities. All 15 participants (100.0%) received intensive treatment via continuous subcutaneous insulin infusion (CSII, insulin pump) during their hospitalization.

In terms of contextual characteristics, participants in all occupational and disease duration subgroups exhibited structured daily routines (either occupation-focused or family care-oriented) and consistent core contextual factors linked to night eating.

### 3.2 Identification of night eating behavior determinants

Utilizing the FBM, manual data extraction systematically identified six primary determinants of night eating behaviors in young and middle-aged individuals with T2DM: motivation, ability, triggers, dietary patterns, psychological factors, and sociocultural influences.

These determinants, detailed in Table 4-1 (Behavioral Drivers, i.e., core initiating factors of night eating) and Table 4-2 (Behavioral Manifestations), further guided the analysis of inter-individual behavioral differences in the cohort.

**Table 4-1:** Behavioral drivers of night eating in young and middle-aged adults with type 2 diabetes mellitus.

Dominant motivation classification	Physiological stress-driven	Emotionally and psychologically driven	Social environment-induced	Health cognitive bias-driven
Qualitative profiling	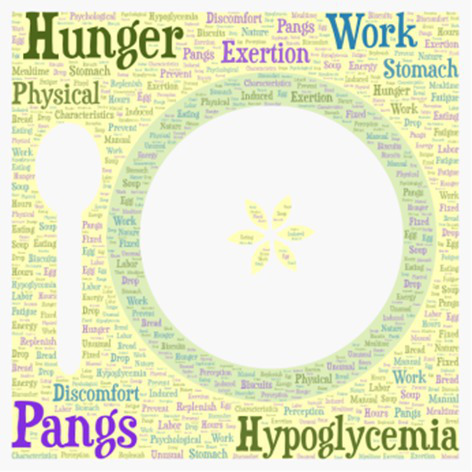	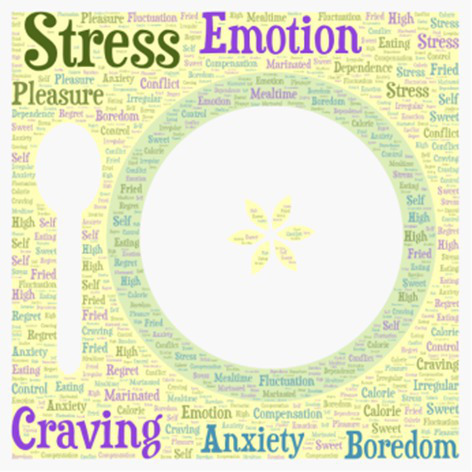	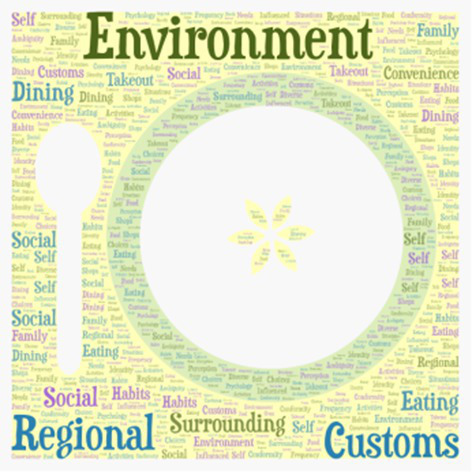	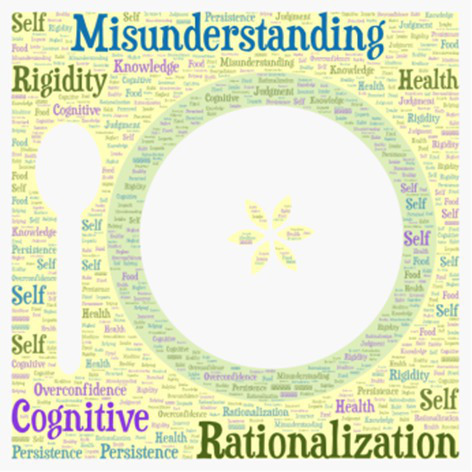
Representative figures	N5、N6、N10、N12、N14	N1、N2、N3、N11	N4、N9、N13、N15	N7、N8
Motivational characteristic**s**	Physiological needs and stress responses originating from hunger	Focus on emotional regulation and psychological compensation	Driven by sociocultural customs and social atmosphere, eating serves social needs	Based on health cognitive one-sidedness, night eating motivation solidified by long-term habits
Capability characteristics				
Food acquisition	Single source, mainly family reserves	Multiple channels (family/dining/takeaway)	Multiple channels (family/dining/takeaway)	Cognitive bias, limitations in food selection ability
Self-management skills Trigger characteristics	Driven by both hunger and the traditional concept, they see no need for control fixed end times, hunger onset times	Uncontrollably but a strong willingness to change external stimuli such as negative mood swings and audio-visual perception	Aware of the harms but struggling to quit, they often counteract it by reducing intake and increasing exercise lively dining atmosphere and social events	Hypoglycemia fearers resist change; cognitive bias sufferers readily modify night eating long-term formation of fixed night eating habits time point

**Table 4-2:** Behavioral manifestations of night eating in young and middle-aged adults with type 2 diabetes mellitus.

Dominant motivation classification	Physiological stress-driven	Emotionally and psychologically driven	Social environment-induced	Health cognitive bias-driven
Dietary characteristics				
Food attributes	Snacks or protein-rich foods	High calorie, heavy taste, strong sensory stimulation (pursuit of instant gratification); nut foods	High calorie, fried (in line with regional food culture/social scene)	Self-considered healthy but unscientific nutritional structure (cognitive misunderstanding)
Meal frequency	High frequency	Higher frequency	Low frequency	Low frequency
Food intake control	Steadily, take satiety as degree	Fluctuation, emotional dominance	Scenario dependence, increased sociability, decreased solitude	Double-fixed diet, habit solidification and do not know its harm
Psychological characteristics				
Physiological dependence	Strong dependence	Weak dependence	Weak dependence	Weak dependence
Emotional or psychologically dependence	Weak dependence	Significant emotional or psychologically dependence	Influenced by people around them, herd mentality occurs	Overconfidence in their diet and health
Health cognition	Cognitive deficit, focusing only on the satisfaction of present physiological needs	Healthy cognitive concept, but difficult to self-regulate eating behavior when emotional fluctuations	Health cognition remains vague, and its potential harm is easily overlooked due to social influence	Fear of hypoglycemia leads to rigid cognition, which is hardly altered even by external persuasion or scientific evidence; in contrast, the other patient shows significant cognitive plasticity
Sociocultural				
Social support	Family members dissuade ineffective	Complex influences, support and opposition coexist	Reinforced by social support	Cognitive bias, social family support difficult to change
Cultural concepts	Traditional “satiety” diet concept bondage	The social culture of “food can relieve emotions” enjoyment and food relief	The dietary customs prevailing in the region	Disconnection between popular science knowledge and individual cognition failure and patient cognition

Interview results revealed significant disparities in night eating behaviors among the young and middle-aged T2DM patients, specifically in the following dimensions corresponding to the identified determinants:

- Motivation sources.
- Food access and preparation ability.
- Trigger timing and types.
- Dietary patterns.
- Psychological and emotional dependence.
- Health awareness.
- Familial, social support, and regional cultural influences.

### 3.3 Characteristics of four night eating behavior subtypes

#### 3.3.1 Physiological stress-driven

During the interviews, five participants reported that night eating behavior was primarily triggered by physiological hunger. One subgroup (N5, N6, N12) experienced excessive energy expenditure due to nighttime work or recreational activities, stating, “The hunger was simply unbearable after going without food for so long.” The other subgroup (N10, N14) had relatively insufficient caloric intake at dinner, expressing, “I did not eat enough for dinner, so I planned to have something more before bed—otherwise, I would easily feel hungry at night.” Physiological hunger thus served as the direct trigger for their night eating behavior.

Regarding food choices, participants N5, N6, and N10 favored non-carbohydrate options (e.g., sugar-free biscuits, egg drop soup) to manage blood glucose, yet clinical monitoring still showed significant fluctuations in their levels. In contrast, participants N12 and N14 preferred takeout, with single nighttime portions comparable to regular dinners.

When discussing the link between night eating behavior and blood glucose, patients commonly believed reducing carbohydrate intake improved glycemic control. For instance, Participant N10 argued small portions of sugar-free cookies posed no negative impact on blood glucose, while Participant N12 acknowledged the risk of fluctuations but confessed to being unable to resist hunger-driven urges to eat at night.

This phenomenon highlights the study population’s distinctive traits: burdened by heavy workloads and irregular schedules, these patients have prominent nocturnal physiological needs. Coupled with limited knowledge of glycemic management, they instinctively prioritize immediate hunger relief over long-term glucose control goals.

Furthermore, despite recognizing the risks of night eating behavior to blood glucose stability, patients struggled to resist hunger-induced physical discomfort. This dilemma underscores the passivity and helplessness of young and middle-aged T2DM patients in self-managing their condition.

Drawing on phenomenological insights from patient experiences and objective monitoring data, this study classifies this behavior as the physiologically stress-driven night eating subtype. Its core characteristics correlate with three factors: hunger sensation, physiological stress response, and circadian rhythm disturbance. Monitoring data showed peak night eating behavior occurred after regular work hours (20:00–24:00) or during the hypoglycemia high-incidence window (02:00–04:00), consistent with the triggering time characteristics in Table 4-1. The mean glucose variability (GV) of the five patients was 31.2% (see Table 3 for raw data), notably exceeding the typical clinical range (< 25%).

Two distinct hunger-induced patterns exist in this subtype: (1) nocturnal hunger from inadequate dinner caloric intake; (2) physiological hunger at night despite post-dinner satiety. Additionally, familial and occupational factors sustain this behavior: although family members disapproved of night eating behavior, patients struggled to change this habit due to strong physiological instincts; the overlap between work stress and post-shift hours may further increase its frequency.

#### 3.3.2 Emotionally and psychologically driven

Interview data revealed that the four patients’ night eating behaviors could be classified into two subtypes based on primary triggers, both predominantly influenced by non-physiological factors. For Patients N1 and N2, the primary trigger was psychological craving. They explicitly stated, “I’m not hungry at all; I just have a craving for food—it’s a psychological desire for food,” underscoring a subjective pursuit driven by their “food-first” values. In contrast, Patients N3 and N11 noted, “Work stress is high, and eating helps relieve pressure,” suggesting night eating as an implicit emotional regulation strategy for stress management.

Two patients (N3 and N2) expressed a clear preference for nut-based foods. The selection of these highly palatable items aligned with their core motivations, further highlighting the notable influence of emotional and psychological factors.

At the subjective experience level, all four patients reported physical and psychological satisfaction after night eating, which further reinforced the persistence of this behavior.

Regarding the link between night eating and blood glucose, Patients N1 and N2 independently recognized this correlation, while Patients N3 and N11 became aware of it only after receiving hospitalization health education. Importantly, this awareness did not alter their night eating behaviors. As Patient N1 candidly put it, “I just cannot help myself; I have to eat,” and Patient N2 stated, “I do not want to restrict my diet.” Ultimately, none of the four patients discontinued night eating. This “cognition-behavior dissociation” primarily stemmed from psychological cravings or emotional stress suppressing rational health cognition, with psychological needs often prioritized over health rationality—highlighting the dominant role of emotional and psychological factors.

Based on in-depth analysis of patients’ lived experiences, this study defined such behaviors as the emotional and psychological driven subtype. Aggregated data (see Table 2) indicated this night eating phenotype was potentially linked to higher BMI and metabolic syndrome, with a more pronounced correlation than other subtypes. Compared to the physiologically stress-driven subtype (see Section 3.3.1), this subtype also exhibited uncontrollable night eating. Differences in behavioral change motivation are outlined in Table 4-1 “Motivation” module: 3/4 patients in this subtype expressed strong willingness to change, versus only 2/5 in the physiologically stress-driven subtype.

#### 3.3.3 Social environment-induced

Interview and aggregated study data (see Table 2) suggest some patients’ night eating may be influenced by social environmental factors. Drawing on patients’ real-life scenarios, four potential exposure pathways were identified: intimate relationships, social interactions, physical spaces, and digital media.

Participant N4 remarked, “My husband gets off work late and has a late-night snack at 9 p.m., usually ordering takeout. When I see him eating, I join in.” This reflects fluke psychology and the psychological permission effect in intimate relationships—instead of physiological hunger or psychological craving, the participant obtained “psychological permission and support” for night eating from shared meals, intuitively highlighting the potential role of intimate coexistence in shaping behavior.

Participant N13 stated, “Friends often invite me to dinner and chat at night, and I find it hard to refuse.” This clearly presents the trade-off between social identity needs and healthy dietary norms; nighttime dinners are more than just eating behaviors, but key ways to maintain social bonds and integrate into circles, emphasizing social identity’s notable influence.

At the physical environment level, Participant N9 noted, “There is a snack street downstairs with 40–50 late-night snack stalls. Sometimes I’m not hungry at all, but I still eat a little.” The accessibility of dense stalls and sensory appeal of food aromas may perpetually stimulate eating desires, disrupting the psychological balance of restrained eating.

Interview data indicated a potential correlation between digital media exposure and night eating: viewing food-related mobile live streams may increase night eating frequency. Multi-dimensional sensory stimulation from visual presentation, immersive experience, and auditory guidance could notably trigger eating urges, acting as a concealed reinforcing factor.

Based on comprehensive analysis of patients’ experiential characteristics, this study tentatively classifies such behavior as the socially environment-induced subtype. Its behavioral triggers (see Table 4-1) depend on external cues (e.g., lively dinners, social invitations) rather than internal signals. Regarding food choices (see Table 4-2), high-calorie and fried foods predominate, aligning with regional food culture and social contexts, with notable potential “herd mentality.” Patients may have vague health awareness and overlook night eating risks due to social influences.

#### 3.3.4 Health cognitive bias-driven

As suggested by interviews and pooled data (see Table 2), the two participants demonstrated cognitive biases in the relationship between disease perception and eating behaviors, exhibiting individual differences in their manifestations. The data objectively documented the logical progression underlying persistent night eating: cognitive bias → behavioral reinforcement → sustained night eating. Furthermore, notable disparities emerged between the two participants regarding their adaptability to behavioral modification.

Participant N7 recounted, “I once had severe nighttime hypoglycemia and now fear such episodes intensely.” N7 further associated hypoglycemia prevention exclusively with increased food intake, remaining unaware of the detrimental effects of high-carbohydrate diets on long-term glycemic control. The participant asserted that this bedtime eating behavior offered a sense of security. It is plausible that the genuine and distressing somatic experience of hypoglycemia shaped the participant’s narrow cognitive framework. Additionally, the participant adopted night eating as a psychological strategy to mitigate the unpredictable risk of hypoglycemia.

Another participant, N8, exhibited a habit of consuming raw peanuts, walnuts, and similar foods at night to achieve “dietary diversity.” N8 held the belief that a healthy diet necessitates a varied food intake; however, they did not connect night eating—regardless of nutritional balance—to metabolic disorders or nocturnal glycemic fluctuations. This suggests that N8’s behavior may arise from a rigid adherence to dietary guidelines, reflecting a one-dimensional cognitive projection and behavioral extension of the concept of “dietary diversity.”

Interview data showed that healthcare providers highlighted this bias to N7, who declined to modify bedtime eating due to concerns about triggering hypoglycemia. In contrast, N8 expressed a willingness to adjust night eating if its negative health effects were substantiated.

Based on the aforementioned experiential analysis, this study classifies night eating influenced by cognitive biases as a subtype of health cognitive bias. Key characteristics include impaired food selection resulting from cognitive biases, the establishment of fixed night eating schedules over time that promote highly rhythmic behaviors, and favorable behavioral plasticity in the absence of significant emotional entanglement.

#### 3.3.5 Age and related factors-stratified analysis of four night eating behavior subtypes

Aggregate study data (see Table 2) documented that participants were stratified into two age groups (18–45 years, *n* = 10; 46–59 years, *n* = 5), alongside stratification by occupational and clinical characteristics (T2DM diagnosis status):

- Newly diagnosed T2DM (defined as disease duration < 1 year): 7 cases (50.0% of total participants), 6 cases in the 18–45 years group, 1 case in the 46–59 years group.
- Previously diagnosed T2DM (defined as disease duration ≥ 1 year): 8 cases (50.0% of total participants), 4 cases in the 18–45 years group, 4 cases in the 46–59 years group. The distribution and core consistency of the four night eating behavior subtypes were recorded.

Further interview and aggregate study data (see Table 2) documented that the core characteristics of all subtypes remained consistent across age, occupational, and clinical groups, with only age- and clinical status-related prevalence differences:

- The physiologically stress-driven subtype (3.3.1) accounted for 4 cases (40.0%) in the younger group and 1 case (20.0%) in the older group, 3 cases (42.9%) in newly diagnosed T2DM patients and 2 cases (25.0%) in previously diagnosed T2DM patients, with consistent triggers of energy deficits from irregular routines (scenarios varying by overtime/skipped meals [younger group] vs. family care-related delayed dinners [older group]) as reported by participants.
- The emotionally and psychologically driven subtype (3.3.2): 3 cases (30.0%) in the younger group vs. 1 case (20.0%) in the older group; 2 cases (28.6%) in newly diagnosed T2DM patients vs. 2 cases (25.0%) in previously diagnosed T2DM patients. This subtype was centered on stress relief, with younger participants reporting work/interpersonal pressures as triggers, and older participants reporting chronic disease anxiety.
- The social environment-induced subtype (3.3.3): 3 cases (30.0%) in the younger group vs. 1 case (20.0%) in the older group; 2 cases (28.6%) in newly diagnosed T2DM patients vs. 2 cases (25.0%) in previously diagnosed T2DM patients. This subtype involved passive social eating, which was recorded more frequently in younger adults, who reported more frequent social interactions.

Interview data additionally documented that the health cognitive bias-driven subtype (3.3.4) was absent in the younger group (0 cases, 0.0%) but present in 2 cases (40.0%) of the older group. It was also absent in newly diagnosed T2DM patients (0 cases, 0.0%) but present in 2 cases (25.0%) of previously diagnosed T2DM patients.

Interview data recorded associated group-specific characteristics:

- Age-related: younger participants reported a focus on immediate needs (hunger, mood) and lower disease awareness; older participants reported longer disease duration and health-conscious eating adjustments (e.g., for blood glucose control).
- Clinical status-related: newly diagnosed T2DM patients reported limited awareness of the association between night eating and blood glucose regulation; previously diagnosed T2DM patients reported more active dietary adjustments for blood glucose control.

## 4 Discussion

### 4.1 Validation of sample characteristics for the reliability of core results

The study sample included 18–59-year-old young and middle-aged adults. Of the 15 interviewees, 11 (73.3%) were employed, 1 retired, 1 student, and 2 homemakers. Regarding disease duration, 46.7% were newly diagnosed with T2DM, and 73.3% had HbA1c ≥ 8.0%. These characteristics align with the clinical subtypes of inpatients in the endocrinology department of tertiary grade A hospitals.

Defined by China’s legal retirement age (≥ 60 years), this cohort has common features of high occupational stress and tight daily schedules, making them more susceptible to night eating, which subsequently worsens glycemic control. This sample composition closely matches the study’s core objective—investigating the intrinsic motivations and driving mechanisms of night eating—and facilitates accurate observation of authentic behavioral patterns unaffected by long-term standardized dietary interventions.

Age- and disease duration-stratified analyses showed that the core driving mechanisms of the four night eating subtypes were consistent across subgroups (differentiated by age, glycemic control, and disease duration), with only variations in prevalence. This confirms the reliability of the core results, with no significant deviation due to the aforementioned sample characteristics.

In-depth analysis of patients’ narratives reveals four stable meaning structures of night eating experiences, which this study summarizes into four subtypes to reflect their inherent subjective diversity.

### 4.2 Physiological stress-driven night eating: glycemic mechanisms and behavioral rigidity

The average glycemic variability (GV) among the five patients in this subtype was 31.2% (see Table 3), which may correlate with reduced nighttime basal metabolic rate, abnormal cortisol secretion, and decreased insulin sensitivity (18). Furthermore, blood glucose fluctuations were strongly associated with patients’ subjective experiences. Patients frequently expressed the conflict of “unbearable hunger prompting eating despite knowing it affects blood glucose”; for instance, N12 remarked, “I know eating late at night is not good, but I cannot help it when hunger strikes.” Even when intentionally selecting non-carbohydrate foods (e.g., sugar-free biscuits, egg drop soup), blood glucose levels could still fluctuate significantly—a phenomenon rarely documented in previous T2DM nutrition-related studies (19). Additionally, patients’ limited understanding of metabolic processes (e.g., gluconeogenesis from protein, glucose conversion from fat) highlights a critical gap in current diabetes education: excessive focus on carbohydrate restriction, with often neglecting protein and fat’s effects on nighttime blood glucose (20).

Moreover, patients often prioritize immediate physiological hunger over long-term blood glucose control risks. This reflects not just a lack of health awareness but a helpless trade-off between acute physiological discomfort and chronic disease management. As N5 noted, “I did not eat any staple food; I cannot just starve.” This aligns with the behavioral economics “present bias” theory (21), where immediate needs overshadow long-term goals, reinforcing night eating behavior. Even aware of long-term risks, patients struggle to alter impulsive choices, revealing marked behavioral rigidity.

Regarding family members’ attitudes toward their night eating, all five patients indicated their families did not oppose it. Instead, they primarily offered verbal reminders to “eat less,” indirectly permitting passive eating and showing tolerance for this type of behavior.

Based on the FBM and interview data analysis, the core contradiction of night eating in this subtype lies in an imbalance of the three FBM elements: motivation from immediate hunger, limited understanding of pertinent metabolic processes, and inadequate family support. This study proposes hierarchical targeted interventions as follows:

Strengthening health motivation: using continuous glucose monitoring (CGM) to visualize blood glucose fluctuation risks, helping patients intuitively understand the potential harms of night eating and enhancing health motivation (22). Since 2019, CGM has been gradually integrated into routine nursing practices of most hospitals, with its efficacy and safety in blood glucose management extensively validated (23, 24).

Popularizing diabetes health literacy among young and middle-aged adults: align with national chronic disease management policies (25), conduct targeted education via offline channels (community family doctors, nurse discharge follow-ups, “Internet+” home services (26)) complemented by online platforms like chronic disease management apps. This addresses health literacy gaps and significantly enhances diabetes health education’s convenience and accessibility.

Optimizing environmental triggers: transform family interventions from mere tolerance to supportive guidance (27) (e.g., assisting in preparing low-GI late-night snacks). Given 73.3% of participants were employed young and middle-aged adults, workplace optimization is also essential (28, 29). Recommend corporate canteens set up low-GI snack supply points, replacing negative dietary triggers with accessible healthy options to help patients avoid passive night eating.

These three components form a closed loop of “motivation arousal—ability support—trigger optimization,” precisely targeting the core mechanism of stress-driven night eating. It compensates for the inadequacy of family intervention under high workplace pressure, aligns with the occupational characteristics of young and middle-aged T2DM patients in this study, and conforms to the goal of multi-level targeted public health interventions.

### 4.3 Emotionally and psychologically driven night eating: coping mechanisms and intervention windows

During interviews, patients often expressed a “psychological craving for food—I just want to eat” and noted that “eating relieves stress.” This connection between eating and immediate emotional relief represents the core phenomenological insight of this subtype. Consequently, it can be inferred that the primary mechanism involves using eating as a means to cope with negative emotions, which aligns with Sambal et al.’s (30) assertion that “eating regulates negative emotions with individual differences.”

This finding underscores the significance of emotional regulation in night eating and indicates that public health interventions should consider individual threshold variations rather than adopting a simplistic “one-size-fits-all” model. In alignment with the study’s original objective of subclassifying night eating to improve intervention precision, it establishes a preliminary mechanistic foundation for future individualized approaches.

In contrast to the findings of Godet et al. (31), which suggest that “negative emotions trigger flavorful, high-energy, low-nutrient food intake,” some patients in this study demonstrated a preference for nuts. This tendency may stem from the unique characteristics of the study population—all were hospitalized patients who received systematic diabetes health education, including brochures, educational videos, and dietary lectures (32). Consequently, these individuals may have developed a foundational understanding of nutrition, prompting them to prioritize high-energy, nutrient-dense foods over flavor-centric, low-nutrient options during episodes of emotional eating. It is essential to recognize that the limited sample size may restrict the generalizability of these findings; however, this data-driven discrepancy provides preliminary insights: emotional eating is not inherently linked to unhealthy dietary choices. Fundamentally, emotional eating addresses the need for emotional regulation, and the nutritional properties of food can serve as a valuable intervention guide.

Based on interview data, the intervention core for this subtype involves balancing emotional needs with healthy dietary choices, rather than imposing a complete restriction on emotional eating. Patients may be encouraged to choose nutrient-dense foods with potential emotional regulatory effects, such as nuts. Tryptophan in nuts—a serotonin precursor—may influence emotion-related neurotransmitters (33). Additionally, the mild flavor and act of chewing can offer psychological comfort, potentially alleviating negative emotions and reducing blood glucose fluctuation risks (34). This approach reconciles psychological coping with metabolic management.

Most patients demonstrated a strong willingness to change during interviews, which aligns with the FBM’s core motivational advantage and may create a critical intervention window. For large-scale public health initiatives, community-led “emotion-diet” group counseling may be more appropriate than individualized Cognitive Behavioral Therapy (CBT), given young and middle-aged patients’ demand for “convenient participation.” Currently, some community health centers provide collaborative guidance from psychologists and dietitians, integrating stress management training (e.g., mindfulness, brief physical activity) to explore breaking the “negative emotions-night eating relief” vicious cycle (35, 36). This model may hold certain promotional value in the future.

### 4.4 Social environment-induced night eating: unique paths in young and middle-aged cohorts

Based on interview findings, some patients’ night eating aligns with Suwalska and Bogdanski (37)—indicating the social environment is a significant determinant of eating behavior. This study offers a unique perspective: participants frequently noted, “I could not resist joining family for meals when they ate appealingly” and “I felt obligated to eat at friend gatherings.” These traits may link to their active social roles (e.g., workplace professionals, family pillars) and strong social bonds (e.g., partner companionship, friend gathering needs), underscoring the uniqueness and complexity of their social environment.

The “socially associated night eating” aligns with Ruddock et al.’s “social facilitation of eating, (38)” which posits individuals consume significantly more with family/friends than alone. Patient experiences show social contexts (e.g., family late-night snacks, friend gatherings) serve as key external triggers, while a strong need for social belonging acts as a crucial intrinsic motivator (39). Collectively, they elicit the automatic “social scenario → night eating” response.

Some patients remarked, “a snack street downstairs makes wandering and eating convenient,” highlighting the physical environment’s impact (food accessibility, temptation) on night eating—consistent with Tonumaipe’a et al. (40) Such cues are explicit external triggers. For young and middle-aged patients with limited self-control, reducing food accessibility (e.g., concealed storage, minimized stockpiling) may prove more effective than verbal advice (41).

Another patient noted, “I could not resist eating after seeing food videos,” aligning with Eroglu et al.’s (42) conclusion that digital media induces night eating. This study further identifies virtual environment penetration as a novel, traditional factor-independent driver. Its correlation with patients’ high digital engagement (43) (e.g., scrolling short videos, watching food live streams before bedtime) amplifies night eating risk. Consequently, interventions disrupting the “environmental trigger chain” may outperform reliance on individual self-control.

Integrating interview data with the FBM, this night eating operates via a “motivation-trigger” dual mechanism: strong social needs as intrinsic motivation, and three key external triggers (social contexts, physical food cues, virtual media exposure). Combined with limited self-control in some patients, these factors may lead to night eating.

This data- and model-based mechanism enables differentiated interventions—moving beyond traditional “individual cognitive education” to an FBM-guided “environment-adapted strategy”: socially driven patients can fulfill social needs via non-dietary activities (e.g., post-meal walks, family board games) while avoiding social dietary triggers; partner-influenced patients may benefit from collaborative couple interventions to optimize family triggers; physically sensitive patients can reduce late-night accessible food reserves at home; media-induced patients may gain from enhanced late-night content supervision and filtering on food apps and short video platforms in the future (44–46).

### 4.5 Health cognitive bias-driven night eating: emotion vs. knowledge gaps

Interview observations suggested that Patient N7’s cognitive bias was closely associated with fear, creating a vicious cycle. In contrast, Patient N8’s cognitive bias arose from misperceived health standards and exhibited low emotional engagement. This divergence in cognitive-emotional linkage may underscore the significant role of emotional intensity in altering cognitive bias-driven night eating behaviors.

In the former case, negative emotions often overshadow motivation, undermining control over night eating, and mere knowledge correction is insufficient to disrupt this cycle. In contrast, for the latter, motivated by rational health needs, individuals may experience a significant reduction in the difficulty of behavioral adjustment upon acquiring accurate information (47).

According to the FBM, this divergence may suggest that, within the study population, emotions primarily regulate behavioral motivation, whereas cognition appears to influence behavioral capacity. This phenomenon (48, 49) may also be associated with the psychological traits of young and middle-aged adults. Although they are susceptible to cognitive biases stemming from incomplete information, these individuals often exhibit a degree of rational judgment and may demonstrate enhanced adaptability in behavioral adjustments when free from negative emotions.

Supported by existing clinical evidence, the “emotion-cognition dual-track intervention” may serve as a comprehensive option for the “fear of hypoglycemia → behavioral rigidity” subtype. Clinically available CGM (50) can provide objective data to alleviate fear, while targeted cognitive correction may assist in disrupting the vicious cycle.

For the pure cognitive bias subtype, the “precision guidance + trigger reminder” strategy may be an effective approach (51). Domestically, Tertiary Grade A hospitals have progressively advanced the development of the National Standardized Metabolic Management Center (MMC) (52). Its standardized intervention process—"cognitive bias identification → personalized guidance → outpatient precise correction”—has preliminarily confirmed the effectiveness of cognitive restructuring and behavioral modification in over 1,000 patients with metabolic diseases, laying a feasible foundation for the strategy’s implementation in primary medical institutions and large-scale promotion. Internationally, trigger reminder models integrating CGM data and mobile APP notifications have shown promising results in correcting night-eating cognitive biases through real-time digital prompts, with their convenience and ease of operation potentially supporting large-scale public health interventions (53).

### 4.6 Core findings and study contributions

To conclude, this study examined a representative population in developing countries—young and middle-aged patients with type 2 diabetes mellitus in China. Given the limited dedicated qualitative studies on night eating behavior in this demographic in China, our research enhanced localized qualitative evidence for night eating in this group.

We innovatively integrated the FBM into the analytical framework, systematically identifying 6 core influencing factors and constructing 4 stable subtypes of night eating behavior. Their core characteristics were consistently validated across subgroups stratified by age and diabetes duration.

This subtype enables healthcare providers to swiftly identify patients’ night eating features upon admission; combined with age stratification, it elucidates each subtype’s distribution. It not only offers scientific targets for precise intervention but also lays a foundational reference for optimizing localized public health management and targeted interventions for T2DM.

Furthermore, this study’s methodology may serve as a valuable reference for profiling inappropriate cognition, attitudes and behaviors in patients with other chronic diseases.

### 4.7 Limitations and future directions

Despite the contributions of this study, several limitations must be acknowledged.

First, concerning the sample, although the four constructed night eating behavior subtypes represent common clinical types, the small interview sample size and recruitment from a single medical institution restrict the generalizability of the results and increase heterogeneity. To mitigate this issue, we have implemented measures such as expanding the general information section to verify the core homogeneity of the population and incorporating an age-stratified analysis to evaluate the stability of the identified subtypes. However, we have not fully eliminated the influence of age-related contextual variations. Notably, the sample primarily comprised hospitalized patients with poor glycemic control, a population particularly relevant to night eating behavior. This clinical context enhances the targeted value of the findings for this subgroup, and the interaction between glycemic control status and night eating subtypes has been elaborated in the discussion to ensure the validity of the results. Furthermore, nearly half of the sample consisted of newly diagnosed cases, which limits the generalizability to the broader young and middle-aged T2DM population. The sample also lacks representation of individuals with specific physiological conditions, such as pregnant or perioperative patients, thereby neglecting the unique night eating patterns influenced by their physiological changes.

Additionally, methodological concerns arise from the potential for subjective interpretation by researchers, which may have affected the results. Moreover, while most patients’ night eating behaviors involve multiple motivations, this study categorized them solely by primary motivation, potentially introducing bias; yet this single dominant profile framework aims to enhance intervention feasibility and scalability by clarifying targeted goals.

Third, due to the small sample size, the assessments in this study were exploratory in nature. Future research will aim to accurately quantify total daily caloric intake and meal-based caloric distribution, while analyzing their interaction with stress levels to eliminate the impact of calorie-related confounding factors. Additionally, standardized nutritional cognition scales will be adopted for quantitative assessment to enhance the reliability of study conclusions. Furthermore, indicators such as daily physical activity duration and intensity will be incorporated to better quantify their correlations with night eating behavior and glycemic metabolism, thereby strengthening the control of confounding factors.

Future research should aim to diversify the sample by including primary hospital patients to represent grassroots populations. It should also incorporate quantitative studies to assess the generalizability of subtypes across broader groups, design group-controlled trials targeting different subtypes to evaluate intervention efficacy, and conduct long-term tracking of how interventions impact patients’ blood glucose fluctuations and quality of life. These initiatives will enhance the theory and practice of chronic disease behavior management, aligning more closely with public health objectives of scalable, population-centered care.

## Data Availability

The original contributions presented in the study are included in the article/supplementary material, further inquiries can be directed to the corresponding author/s.
